# Consequences of Essential Fatty Acids

**DOI:** 10.3390/nu4091338

**Published:** 2012-09-24

**Authors:** Bill Lands

**Affiliations:** 6100 Westchester Park Drive, Apt. 1219, College Park, MD 20740, USA; Email: wlands@mail.nih.gov; Tel.: +1-301-345-4061

**Keywords:** arachidonic, bioactive mediators, cardiovascular, desaturase, eicosanoids, healthcare claims, highly unsaturated fatty acids (HUFA), immune-inflammatory, leukotrienes, linoleic acid, omega-3 (*n*-3), omega-6 (*n*-6), prostaglandins, polyunsaturated fatty acids (PUFA), selective receptors, signal transduction

## Abstract

Essential fatty acids (EFA) are nutrients that form an amazingly large array of bioactive mediators that act on a large family of selective receptors. Nearly every cell and tissue in the human body expresses at least one of these receptors, allowing EFA-based signaling to influence nearly every aspect of human physiology. In this way, the health consequences of specific gene-environment interactions with these nutrients are more extensive than often recognized. The metabolic transformations have similar competitive dynamics for the *n*-3 and *n*-6 homologs when converting dietary EFA from the external environment of foods into the highly unsaturated fatty acid (HUFA) esters that accumulate in the internal environment of cells and tissues. In contrast, the formation and action of bioactive mediators during tissue responses to stimuli tend to selectively create more intense consequences for *n*-6 than *n*-3 homologs. Both *n*-3 and *n*-6 nutrients have beneficial actions, but many common health disorders are undesired consequences of excessive actions of tissue *n*-6 HUFA which are preventable. This review considers the possibility of preventing imbalances in dietary *n*-3 and *n*-6 nutrients with informed voluntary food choices. That action may prevent the unintended consequences that come from eating imbalanced diets which support excessive chronic actions of *n*-6 mediators that harm human health. The consequences from preventing *n*-3 and *n*-6 nutrient imbalances on a nationwide scale may be very large, and they need careful evaluation and implementation to avoid further harmful consequences for the national economy.

## 1. Introduction

**Figure 1:**
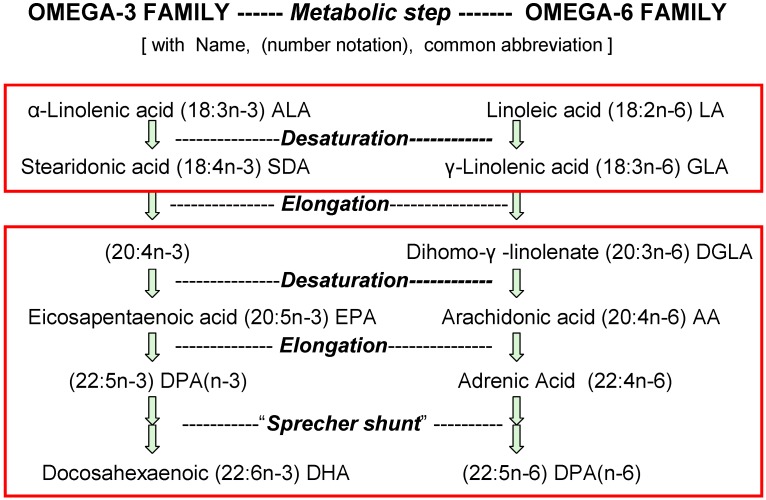
Competition between *n*-6 and *n*-3 forms of essential fatty acids (EFA). Typical daily amounts of these acids eaten in the USA as percent of food energy (en%) are for 18-carbon EFA 7% *n*-6 acids (18:2*n*-6 + 18:3*n*-6) and 1% *n*-3 acids (18:3*n*-3 + 18:4*n*-3), but for 20- and 22-carbon HUFA they are only 0.08% *n*-6 acids (20:3*n*-6, 20:4*n*-6, 22:4*n*-6 + 22:5*n*-6) and 0.04% *n*-3 acids (20:5*n*-3, 22:5*n*-3 + 22:6*n*-3).

The discovery and identification of vitamins and essential nutrients often begin by measuring growth and development of infant laboratory animals. For essential fatty acids (EFA), such studies started eighty years ago [1] with identification of vitamin-like properties of linoleic acid (18:2*n*-6) and linolenic acid (18:3*n*-3). Later studies showed that the elongated and desaturated homologs shown in Figure 1 also support growth (e.g., [2]). Now, we know that the consequences of *n*-3 and *n*-6 nutrients for humans go far beyond the support of healthy growth of infants which is achieved with intakes of linoleic acid less than 0.5% of food energy (en%) [3,4].

The list of human health problems implicated in relative deficits of *n*-3 nutrients with excessive actions of *n*-6 bioactive mediators has grown to include heart attacks [5], atherosclerosis, thrombosis [6], arrhythmia, stroke, immune-inflammatory disorders [7], asthma, arthritis, cancer proliferation [8], obesity [9], psychiatric disorders, depression, suicide, homicide [10,11], oppositional behavior, unproductive workplace behaviors, length of stay in hospitals [12] and annual healthcare claim costs [13]. The consequence of this broad impact on health is that treatments of the EFA-based events have created a major multi-trillion dollar part of the USA economy. The National Library of Medicine now lists over 12,800 review articles about just the eicosanoids that are derived from the 20-carbon essential fatty acids. The biomedical literature has much information to help people identify and prevent the current imbalanced intakes of *n*-3 and *n*-6 nutrients that cause preventable clinical disorders and continue escalating healthcare costs. 

Health and disease are consequences of environmental conditions interacting with genetic propensities. Neither alone is the sole controller of health. In this review, two important parts of the environment are the external supply of essential nutrients in available foods and the internal supply of food-derived intermediates that accumulate in our cells and tissues. What we eat is under our voluntary control and is more easily altered than our genome. However, once *n*-3 and *n*-6 derivatives accumulate in tissues [14], they can form bioactive mediators which act differently at specific receptors present on nearly every cell and tissue in the human body [15]. Many gene-defined enzymes and receptors favor actions by *n*-6 mediators and allow the accumulated proportions of *n*-3 and *n*-6 intermediates in our tissues to affect almost every physiological and pathophysiological process in human life. 

The proportions of *n*-3 and *n*-6 mediators accumulated in the inner environment of our cells and tissues reflect quantitatively those in the foods we eat from our external environment [14]. However, the *n*-3 and *n*-6 homologs have different dynamics in the formation and action of bioactive mediators [15]. As a result, preventable health disorders are unintended consequences from eating imbalanced amounts of the two types of EFA [6,7]. The large annual financial losses from these food-based health disorders might be prevented if they were better monitored, understood and prevented. 

When the nutrient imbalance is prevented, people are healthy and some treatment costs are not needed. Rather, the funds that currently flow in the USA from the employers and employees who pay for the costs of preventable healthcare claims might be diverted to fit other priorities. While not all members of society will welcome preventive nutrition changes, the large consequences of the nutrient imbalances must be openly addressed. This review considers first the contrasting dynamics of two multi-step pathways by which dietary EFA produce preventable health consequences. Understanding those dynamics helps design effective preventive measures.

## 2. Non-Selective Competitions When Converting *n*-3 and *n*-6 Nutrients into Tissue Esters

Enzymes that catalyze the metabolic steps of digestion, absorption, transport, desaturation, elongation, esterification and remodeling of *n*-3 and *n*-6 nutrients have genetically defined affinities for substrates based on chain length as well as the number and location of double bonds. However, none of these enzymes appreciably discriminate between the *n*-3 and *n*-6 structures (Figure 1). The chemical features that contribute to affinity of fatty acids for the enzymes that handle them is much more complex than indicated by terms in general daily use [16]. The enzyme selectivities are not explicitly defined by the terms “saturated”, “unsaturated”, “polyunsaturated” (PUFA) and “highly unsaturated” (HUFA). For example, acyltransferases that preferentially esterify HUFA to the 2-position of membrane phospholipids have unexpected selectivities that seem to detect “pi bonds” and precise double bond locations along the carbon chain [16]. However, their inability to discriminate between *n*-3 or *n*-6 structures [17] allows the relative abundances of these substrates to affect the relative abundances that are accumulated in tissues. Figure 1 shows the metabolic steps that convert 18-carbon nutrients to the 20- and 22-carbon HUFA. 

A consequence of indiscriminate action by the enzymes moving EFA from foods into tissues is that there is no inherent “optimal” or “biological” *n*-3 and *n*-6 proportion maintained in tissues. Rather, the proportions of *n*-3 and *n*-6 in 20- and 22-carbon highly unsaturated fatty acids (HUFA) that accumulate in tissue lipids have a predictable competitive hyperbolic relationship with amounts of *n*-3 and *n*-6 18-carbon acids eaten. The mid-point of nutrient effectiveness for HUFA accumulation and for growth was near 0.1% of food calories [6,18,19]. The legend to Figure 1 notes that current daily intakes by Americans are very much greater than that.

As 18-carbon *n*-3 or *n*-6 acids increase in the diet, their competition with endogenous *n*-7 and *n*-9 acids for desaturation and elongation makes 20:4*n*-7 and 20:3*n*-9 become a smaller proportion of tissue HUFA [6,18,19]. Some people regard with alarm the presence of any 20:3*n*-9 in blood lipids as a sign of lower than intended EFA intakes. However, dietary intakes of 18:2*n*-6 near 1 en% prevent physiological deficits and cause the proportion of *n*-9 in HUFA to be near 10% [18]. Parenteral feeding studies confirmed that no clinical deficiency signs occur whenever *n*-6 HUFA in blood was greater than *n*-9 HUFA [20]. Thus, the frequently observed absence of detectable *n*-9 HUFA in blood may actually be a sign alerting us to excessive intakes of competing *n*-3 and *n*-6 acids [6]. Constructive evaluations of this competitive consequence may lead people to consider how to estimate whether their personal voluntary *n*-3 or *n*-6 EFA intakes are enough or too much [6]. Either interpretation will likely have financial consequences that will stimulate further comment from food marketers.

### 2.1. Remodeling Reactions with Membrane Phospholipids

Tissue phospholipids undergo a continual remodeling process that allows cellular membranes to fairly rapidly accumulate HUFA at the 2-position [16]. Like elongation and desaturation reactions, the acyltransferases [16] that esterify EFA to membrane phospholipids and the phospholipases [21] that hydrolyse them discriminate among chain lengths and double bonds but not the *n*-3 and *n*-6 structures. The rapid turnover and non-selective accumulation of HUFA creates a condition in which whole blood, plasma, serum and erythrocytes acquire similar proportions of *n*-3 and *n*-6 in the mixed HUFA [22,23,24,25]. Gas chromatographic analyses measure the fatty acid contents in both the external environment of foods and the internal environment of tissue phospholipids. Several different ways of expressing those contents have been used for health risk assessment [25]. Each may have a different utility for different interpretations. This review uses en% or mg/Cal for EFA in foods from external environment [26] because it has a useful predictive relationship to the %*n*-6 (or *n*-3) in HUFA in the internal environment [27]. Further, the %*n*-6 in blood HUFA is a valid surrogate for clinical endpoints in preventive nutrition interventions [5,6,13]. 

Three enzymes, cytosolic PLA2 (cPLA2), Ca^2+^-independent PLA2 (iPLA2) and secretory phospholipase A2 (sPLA2) all hydrolyze both *n*-3 and *n*-6 HUFA from the *sn*2 position of the major types of phospholipids. The cPLA2 preferentially acts on longer acyl chains with more double bonds (HUFA > PUFA > monounsaturated acids) [28]. With phosphatidyl choline (PC) as substrate, the fatty acid preference of cPLA2 is arachidonic (20:4) > linolenic (18:3) > linoleic (18:2) > oleic (18:1) > palmitoleic (16:1) [29]. Although this order of preference is often termed “specific for arachidonic acid” [30], evidence for the enzyme preferring 20:4*n*-6 to 20:5*n*-3 is still not available. The order of preference among PCs containing 20-carbon *sn*-2 acyl chains was arachidonic (20:4) > dihomogammalinoleic (20:3) > eicosadienoic (20:2) > eicosenoic (20:1) > eicosanoic (20:0), and there appeared to be a preference for positional isomers with double bonds closest to the *sn*-2 ester bond (eicosatrienoic 5,8,11 > 5,8,14 > 5,11,14 > 8,11,14), however acids with a double bond at position 4 were not tested [29]. 

Paradoxically, cPLA2 hydrolytic action on phosphatidyl ethanolamine substrates decreased dramatically with chains longer and more unsaturated than arachidonic acid (20:4) the order being arachidonic (20:4) > pentaenoic (20:5) > docosahexaenoic (C22:6) [31]. Because cPLA2 binds with high affinity to phospholipids with 22:6*n*-3 (DHA), the less easily hydrolyzed DHA phospholipids are actually competitive ligands that slow the hydrolytic release of the abundant alternate substrate, 20:4*n*-6 [31]. 

The consequence of binding a competing slow substrate (or weak agonist) is that it becomes an antagonist that slows action with the faster substrate or agonist. This basic principle in pharmacology is seen for many of the selective protein-ligand interactions in Section 3 of this review. Another basic principle is that signaling actions are often terminated by removing the active agent or by desensitizing or downregulating the receptor at which it acts. For example, the released HUFA that could form bioactive signaling agents are removed by forming acyl-CoA thiol esters that re-esterify the acyl chain to phospholipids [32]. Rapid re-esterification limits HUFA availability to the enzymes that form signaling mediators, making the signaling event even more transient as the system returns to a “resting” state.

### 2.2. The External Environment Affects the Internal Environment

Major differences in *n*-3 and *n*-6 proportions of tissue HUFA have been reported for diverse ethnic groups. The proportions seem to not be determined appreciably by gene-defined enzymes selecting *n*-3 or *n*-6 structures [6,14]. Rather, the proportions of *n*-3 and *n*-6 HUFA in tissues are primarily determined by the relative amounts of competing *n*-3 and *n*-6 nutrients that are eaten [6,14,33]. A quantitative empirical hyperbolic equation reliably predicts the impact of dietary EFA on tissue HUFA [6,33]. To use this information to design effective controlled clinical studies, the equation was embedded in a spreadsheet [34] that uses the en% of four types of nutrients shown in Figure 1. It gives useful estimates of the HUFA proportions likely maintained in tissues when eating those amounts. Differences in the EFA eaten reliably predicted the observed differences in the *n*-3 and *n*-6 proportions of blood HUFA for 92 different study groups in eleven different countries [27]. The very wide range of ethnic food habits worldwide causes the wide range of blood HUFA proportions (28% to 88% *n*-6 in HUFA) that has been reported for different populations [5,7,14]. 

Epidemiologists showed major differences in accumulated HUFA for populations with similar genetics and different food choices, such as Japanese living in Japan [35,36] or Hawaii and California [37]. Also, people in countries that once ate foods described as a “traditional Mediterranean diet” had a low prevalence of cardiovascular disease. They now have a higher prevalence of myocardial infarction [38] as global international food marketing changes traditional food availability and creates misunderstandings about what is “healthy eating”. The situation provides another example of unintended consequences from altering the supply of dietary *n*-3 and *n*-6 fats without adequate attention to the consequences it has on human health. More explicit awareness of the actual *n*-3 and *n*-6 nutrients in foods would allow consumers to make voluntary corrective nutrient choices.

Recently, the balance among eleven *n*-3 and *n*-6 nutrients in typical daily food items was expressed by an explicit Omega 3–6 Balance Score that compresses the balance into a single value for each of over 5100 food items [26]. Average daily food Scores for worldwide food habits appear to range from +3 to −8. Foods with more positive Omega 3–6 Balance Scores will increase the percent of *n*-3 in blood HUFA and those with more negative Omega 3–6 Balance Scores will increase the percent of *n*-6 in blood HUFA. Detailed compositions of foods provided in the USDA Nutrient Database [39] were converted to Omega 3–6 Balance Scores for 5100 foods and posted on a website [40]. The information can be downloaded and used as an “app” for mobile devices [41] to aid food choices when shopping or planning meals. The calorie-weighted average scores for typical daily menus relate linearly to the *n*-6 in blood HUFA [26]. In this way, the likely impact of the external food environment on the inner tissue environment can be estimated and altered when desired. 

Proportions of 30% to 40% *n*-6 in HUFA are associated with eating traditional Japanese foods that have an average daily menu balance near +1 [26]. In contrast, a higher value of 60% *n*-6 in HUFA is associated with the traditional Mediterranean diet that has an average Balance Score near −3. A value of 78% *n*-6 in HUFA is associated with the current American diet [26,27] with an average Balance Score near −6 or −7. The observed proportion of *n*-6 in HUFA is strongly associated wigth the incidence of cardiovascular disease in these populations [5].

### 2.3. Impact of Dietary 18-Carbon EFA

Typical daily intakes in the USA include 1100 mg *n*-3 and 15,000 mg of *n*-6 18-carbon EFA (18:3*n*-3 plus 18:4*n*-3 and 18:2*n*-6 plus 18:3*n*-6, respectively) with 110 mg *n*-3 and 185 mg *n*-6 HUFA (20:5*n*-3, 22:5*n*-3 plus 22:6*n*-3 and 20:3*n*-6, 20:4*n*-6, 22:4*n*-6 plus 22:5*n*-6, respectively). The abundant 18-carbon nutrients are about 7-fold less effectively accumulated into tissue HUFA than are the dietary HUFA nutrients [26]. However, the very much greater abundance of the 18-carbon nutrients lets them dominate the overall Omega 3–6 Balance Food Score and the resulting %*n*-6 in blood HUFA. Eating equal amounts of *n*-3 and *n*-6 18-carbon nutrients would give a lower %*n*-6 in HUFA and likely provide a more benign health consequence.

Discovery of genetic differences in the 5- and 6-desaturases that convert 18-carbon nutrients to HUFA in humans provided an informative example of how competing dietary supplies in the external environment affect the internal tissue HUFA. When the enzymes have faster rates of forming HUFA, the proportions of *n*-3 and *n*-6 18-carbon nutrients have more impact on the accumulated tissue HUFA. For people eating a “modern” diet, those with more active FADS haplotypes accumulate higher proportions of *n*-6 acids in HUFA and have higher systemic inflammation and greater risk of coronary artery disease [42]. People with haplotype D have a faster rate of biosynthesis of both *n*-3 and *n*-6 HUFA from 18-carbon dietary precursors [43]. This harmful consequence seems not so much due to preferential metabolism of the *n*-6 homologs as it is due to the high proportion of *n*-6 18-carbon EFA in diets which are then effectively and non-selectively transferred into tissue HUFA.

The very high frequency of haplotype D in Africa (99%) and the high linkage disequilibrium in the FADS region suggest that this part of the human genome once had “survival value” and has been subjected to positive selection [43]. Efficient formation of HUFA by haplotype D may have had value in the prehistoric habitat where the dietary 18-carbon acids in vegetation had balanced amounts of *n*-3 and *n*-6 nutrients and HUFA were not easily available in foods. However, eating “modern” foods that have much more *n*-6 than *n*-3 fats makes a health risk from rapid conversion of dietary 18-carbon *n*-6 acids into tissue HUFA. Of the top 100 key foods in the USA diet, ten food items have high contents of 18:2*n*-6 and much more negative Omega 3–6 Balance Scores (from −50 to −24) than the overall daily average near −6 [26]. People can not depend on the FADS enzymes to “correct” such an imbalance in dietary EFA. The evidence now available indicates that the current USA food environment provides intakes of 18:2*n*-6 high enough to have serious health and financial consequences for people with haplotype D. 

## 3. Selective Conversion of HUFA into Transient Signaling Actions

In contrast to the proteins described in Section 2, many of the proteins that convert tissue HUFA into transient bioactive mediators discriminate between the *n*-3 and *n*-6 structures. The EFA-based mediators include endocannabinoids, prostaglandins, leukotrienes, epoxides, lipoxins and other active lipids. The prostaglandins and leukotrienes are formed by several sequential steps in which dynamic interactions affect the intensity of the hormone-like signals. The steps include: stimulated release of precursors from 2-acyl esters of phospholipids, oxidation to intermediate forms, rearrangement of intermediates into active, evanescent bioactive mediators plus selective binding and transient actions of the mediators at cellular receptors which transduce signals into the cell via G-protein coupled actions [15,44]. At all stages, the dynamics of signaling depend on a balance between the formation and removal of active intermediates. 

**Figure 2:**
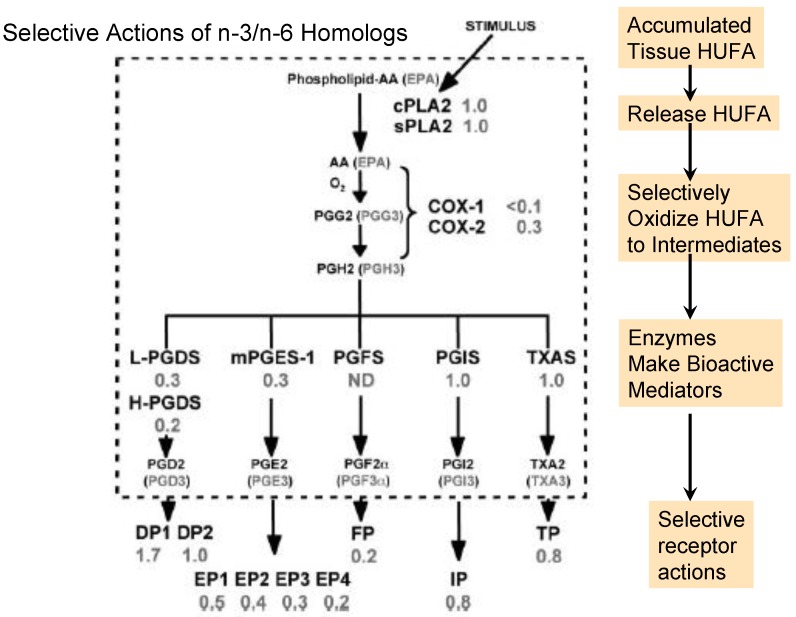
Selective events for *n*-3 and *n*-6 homologs during prostaglandin formation and action. The number beside each event shows the efficacy of the *n*-3 homolog relative to the *n*-6 homolog. Abbreviations for the enzymes and receptors are: cPLA2, cytosolic phospholipase A2; sPLA2, soluble phospholipase A2; COX-1, cyclooxygenase-1; COX-2, cyclooxygenase-2; L-PGDS, lipocalin prostaglandin D synthase; H-PGDS, hematopoietic prostaglandin D synthase; m-PGES-1, microsomal prostaglandin E synthase-1; PGFS, prostaglandin F synthase; PGIS, prostaglandin I synthase; TXAS, thromboxane synthase; DP, prostaglandin D receptors (1–2); EP, prostaglandin E receptors (1–4); IP, prostaglandin I receptor; TP, thromboxane receptor. The figure is modified from that published by Wada *et al.* [15].

In contrast to the events in Figure 1, the gene-defined selective processes in Figure 2 tend to provide more vigorous and intense actions with *n*-6 than *n*-3 homologs [15]. Although the overall set of events in Figure 2 is often called the “arachidonic acid cascade”, eicosapentaenoic acid produces a corresponding set of *n*-3 homologs (noted in parentheses). The *n*-6-mediated actions in inflammatory and thrombotic events support a multi-billion dollar market in aspirin-like non-steroidal anti-inflammatory drugs that inhibit COX-1 and COX-2 and moderate unwanted EFA-derived consequences. However, to slow excessive signaling by *n*-6 mediators, we may choose either to lower tissue proportions of *n*-6 in HUFA with competing *n*-3 HUFA or to design more effective drugs. The wide range of consequences that EFA-based signals have on human health and behavior is illustrated by a few examples in the following section. 

### 3.1. Stimulated Release of Tissue HUFA

Activation of cPLA2 by a transient rise in intracellular Ca^2+^ is often the first step of converting tissue HUFA esters to the non-esterified form that can then form active hormone-like eicosanoids as noted in Figure 2. The high proportion of linoleic acid among the EFA of average American foods (see Figure 1 legend) relative to foods from other places maintains arachidonic acid at relatively high proportions in the HUFA of tissue phospholipids [6,26,27]. However, deliberate food choices can raise the % *n*-3 HUFA and lower the proportion of arachidonate in tissue HUFA available for subsequent transformations in the “arachidonate cascade”. Throughout the chain of events that produces physiological consequences from bioactive forms of essential fatty acids, the strength of the physiologic consequence depends on the relative rate of formation and removal of each intermediate form. The rate of release from cell membrane phospholipids is counterbalanced by the local rate of forming the CoA ester and re-esterifying the intermediate acid back into tissue lipids as noted in section 2.1. The transient balance between signaling and not signaling prevents physiologic events from becoming chronic pathophysiologic processes. 

### 3.2. Oxidation to Intermediate Forms

Three types of oxidative enzymes convert HUFA into intermediates that form important signaling agents: cyclooxygenase (COX-1 and COX-2), lipoxygenase (5-LO, 12-LO, 15-LO) and cytochrome P450 (epoxygenase and hydroxylase). The first product of the COX reaction is a hydroperoxide, PGG, that rapidly forms another short-lived form, PGH. COX-1 reacts much more rapidly with the *n*-6 arachidonate to form PGH2 than with the *n*-3 eicosapentaenoate to form PGH3 [15]. COX-2 also reacts several-fold faster with the *n*-6 than *n*-3 substrate. This selective preference for forming *n*-6 PGH2 ensures a vigorous supply of this intermediate for the different cellular prostaglandin synthases to form *n*-6 rather than *n*-3 bioactive mediators. Selective action of *n*-6 mediators beyond what is needed for normal physiology creates clinical concerns about chronic health disorders [44]. 

The intense formation and action of *n*-6 mediators is lowered when a tissue accumulates more abundant *n*-3 HUFA compared to *n*-6 HUFA. Slow formation of the *n*-3 active agents combined with their rapid removal by inactivation makes signaling by *n*-3 mediators much less vigorous than occurs with *n*-6 homologs. The transient nature of eicosanoid signaling is ensured by rapid actions of the abundant 15-hydroxy dehydrogenase. This enzyme gives rapid inactivation of all of the diverse active prostaglandins in Figure 2 and prevents their action at cellular receptors. Over-expression of 15-PGDH counteracted the oncogenic action of PGE2 and made cancer cells susceptible to apoptotic death [45]. 

### 3.3. Rearrangement of PGH into Active Eicosanoids

PGH2 and PGH3 react with diverse prostaglandin synthases to form many different eicosanoids, PGD, PGE, PGF, PGI, TXA (thromboxane), that bind to specific receptors and produce specific actions. Alternatively, the PGH can diffuse to nearby cells where a different set of synthases in that location can convert it to alternate active eicosanoids. Such transcellular diffusion of PGH allows a nearby cell to form eicosanoid signals in the absence of mobilizing its own phospholipase or cyclooxygenase activities [46]. For example, PGH formed in platelets interacts with platelet TXA synthase to form thrombogenic TXA, whereas it can also diffuse into vascular endothelial cells where endothelial PGI synthase converts it into anti-thrombotic PGI. The two different synthases do not appreciably discriminate between the *n*-3 and *n*-6 structures, but the selective receptors give different physiological responses. 

Two different PGD synthases (H-PGDS, L-PGDS) react several-fold more rapidly with PGH2 than PGH3. Similarly, an inducible prostaglandin E synthase (PGES-1) reacts several-fold faster with the *n*-6 than the *n*-3 PGH [15]. The consequence of both synthases acting in concert with COX-2 ensures a much more vigorous action of PGD2 and PGE2 compared to PGD3 and PGE3. Two other synthase isoforms, cytosolic PGES and PGES-2, form PGE with a selectivity not yet known.

The selectivity of PGF synthase for *n*-3 and *n*-6 structures is not known, but the G-protein coupled transmembrane PGF receptor, FP, binds and transmits a signal much more vigorously with the *n*-6 PGF2 than the *n*-3 PGF3 [15]. A strong FP signal is likely the basis for the discomfort of dysmenorrhea, which is diminished greatly by slowing COX action with non-steroidal anti-inflammatory drugs [47]. The combined selective effect of COX and FP favoring *n*-6 signaling actions may lead to a milder FP action when tissue HUFA have equal amounts of *n*-3 and *n*-6 precursors compared to the high proportion of *n*-6 in HUFA typical for Americans. Similarly, undesired FP actions in pre-term labor [48] are likely milder when tissues produce less *n*-6 PGF2 and more *n*-3 PGF3. There is a clear chain of events linking the *n*-3 and *n*-6 nutrients in the external environment to the intensity of health-impairing events in tissues.

### 3.4. Endocannabinoid Signaling

An alternate path of eicosanoid signaling follows transient activation of phospholipase C (PLC) rather than phospholipase A. PLC releases diacylglycerols that are hydrolyzed by diacylglycerol lipase (DAGLα or DAGLβ) [49]. The process can form an active endocannabinoid intermediate, 2-arachidonyl glycerol (2-AG) which binds and activates CB1 and CB2 cannabinoid receptors. Activation of CB2 receptors on mast cells has beneficial antiinflammatory effects by decreasing the mast cell release of pro-inflammatory mediators. Also, activation of CB1 receptors on bronchial nerve endings has beneficial bronchodilator effects on airway smooth muscle important in airway hyperreactivity and asthma [50]. 

The transient availability of the bioactive mediator, 2-AG, is limited by monoacylglycerol lipase (MAGL) which hydrolyses the active agent to non-esterified HUFA [51] which may then be converted to active eicosanoids that have effects other than the endocannabinoids. Little is known about the selectivity of DAGL and MAGL for *n*-3 and *n*-6 structures. An excellent review of the evidence for DAGL-dependent endocannabinoid signaling during axonal pathfinding, synaptic plasticity and adult neurogenesis was provided by Oudin *et al.* [49]. 

Another EFA-based endocannabinoid that activates the endocannabinoid receptors is *N*-arachidonoylethanolamide, anandamide. The selective reactions forming and hydrolyzing this active mediator remain inadequately studied, and the degree to which *n*-3 and *n*-6 homologs of anandamide occur and influence tissue metabolism needs more explicit answers [52,53]. If no *n*-3 homolog is formed in tissues, a hitherto unknown selective enzyme remains to be discovered. The growing realization of a regulatory role for endocannabinoids in appetitive behaviors and energy homeostasis [54] was strengthened by recent evidence for dietary *n*-6 linoleic acid stimulating obesity [9]. Further study may clarify if the effect that shifts metabolism toward fat accumulation acts directly on appetite or on metabolic fuel allocation. 

### 3.5. Selective Binding and Signaling at Cellular Receptors

The wide impact of EFA on daily life becomes more evident when we examine the many eicosanoid receptors and their G-protein coupled signals that affect muscle, brain and immune cell functions. The consequences of the diverse actions of the G-protein coupled receptors have been studied intensively, especially by using genetic knock-out mice that lack specific prostanoid receptors [55,56]. The PGD receptor, DP1, appears to be one of the few proteins that interacts more strongly with *n*-3 than *n*-6 structures [15]. In contrast, another receptor, DP2, handles both *n*-3 and *n*-6 structures equally. The eicosanoid receptors can be classified by their actions on G-protein mediated intracellular signaling paths [56]. Contractile receptors (EP1, FP, and TP) couple to Gq and raise intracellular Ca^2+^. They also activate the prenylated pro-inflammatory and proliferative factor, Rho, by way of G12/13. Relaxant receptors (DP, EP2, EP4, IP) raise intracellular cAMP levels, and EP4 also activates PI3K which then activates signaling by Akt (protein kinase B). The inhibitory receptor (EP3) couples to Gi and lowers intracellular cAMP levels. All four different PGE receptors create more intensive signals with the *n*-6 PGE2 compared to the *n*-3 PGE3 [15]. Their selective location on different cells makes an amazing array of diverse physiological responses to stimuli.

For example, illness-induced fever is mediated by a pathway of gene-defined enzymes and receptors: interleukin-1 induces COX-2 that forms PGH2 which PGES converts to PGE2 that acts on the EP3 receptor which activates Gi-mediated lowering of intracellular cAMP levels. Alternatively, illness-induced anorexia is mediated by PGE2 acting on EP4 receptors of histaminergic neurons [57]. The anxiogenic effect of repeated social defeat (subjugation or frustration) is mediated by a signaling pathway of COX-1 forming PGH2 that PGES converts to PGE2 which then activates an EP1 receptor which activates Gq-mediated elevation of intracellular Ca^2+^. Genetic deletion of any of the mediating proteins prevents induction of social avoidance behavior that comes from repeated social defeat [58]. The proteins act less vigorously with the *n*-3 than *n*-6 homologs, making the lowering of dietary and tissue proportions of *n*-6 relative to *n*-3 EFA a possible way to moderate unwanted behaviors. Another example of EFA-derived signaling in the central nervous system is an anxiolytic action of PGE2 on EP1 and EP4 receptors [59]. Because genetic deletion of EP4 abolished the anxiolytic effect of PGE2, the EP4 receptor must play a dominant role in this condition. The PGE2-induced anxiolytic-like activity depended also on serotonin 5-HT-1A, dopamine-D1 and GABA-A receptor signals. 

### 3.6. Lipoxygenase and Leukotriene Formation and Action

The lipoxygenase-induced oxidative formation of *n*-3 and *n*-6 leukotriene A (LTA5 and LTA4, respectively) appears to be fairly non-selective for *n*-3 and *n*-6 structures. Like PGH, LTA is an evanescent intermediate in a pathway for forming bioactive mediators [60]. It can diffuse to adjacent cells where synthases may form active mediators, either LTB or LTC, LTD or LTE [46]. The active mediators, in turn, may diffuse and bind to and activate cell-surface receptors on the same or adjacent cells. A selective response of the receptors to *n*-3 and *n*-6 structures allows different proportions of dietary *n*-3 and *n*-6 nutrients to influence many different immune-inflammatory processes. The receptor for LTB responds vigorously to the *n*-6 homolog, making LTB4 one of the most potent chemotactic agents for recruiting neutrophils and macrophages and creating inflammatory conditions in a tissue. Ionophore-activated neutrophils non-selectively generate LTB4 and LTB5 in a ratio that corresponded to the proportions of AA and EPA in the membrane phospholipids. Added EPA competitively attenuated the generation of LTB4 as it was converted to the weak partial agonist, LTB5 [61]. 

A major clinical impact of the *n*-6 leukotriene, LTB4, comes from the very selective interaction at the BLT receptor. The *n*-3 mediator, LTB5, was approximately 100 times less potent than the *n*-6 LTB4, in enhancing complement receptor responses and in eliciting chemotaxis of leukocytes, and it was 10,000 times less potent in releasing lysozyme from human neutrophils [62]. Added *n*-3 EPA suppressed in a competitive, dose-dependent manner the lung vascular leakage induced by a bacterial toxin [63]. This competition was accompanied by a blocked generation of *n*-6 leukotrienes and a dose-dependent increase in *n*-3 leukotrienes: LTB5, LTC5, and LTE5. Added 20:5*n*-3 competitively shifted leukotriene generation from the *n*-6 to the *n*-3 homolog as it fully antagonized 20:4*n*-6-induced amplification of lung leakage. This competition illustrates an important event whereby food choices impact immune-inflammatory events.

Obese mice lacking the LTB receptor, BLT-1, were protected from systemic glucose and insulin intolerance associated with a decrease in inflammation in adipose tissue and liver and a decrease in hepatic triglyceride accumulation [64]. A loss of insulin signaling in liver and skeletal muscle comes from excessive pro-inflammatory signals from a LTB4-stimulated accumulation of macrophages in the tissues. Ironically, pharmacologic attempts to inhibit the LTA hydrolase that forms pro-inflammatory LTB4 may also prevent LTA hydrolase from removing a different proinflammatory agent from the inflamed site [65]. The situation may be one in which preventive nutrition by eating foods with more positive Omega 3–6 Balance Scores may succeed better than drug inhibition of the hydrolase. 

### 3.7. Other Active Eicosanoid Signaling Agents

In contrast to immune-inflammatory events driven by LTB, LTC, LTD or LTE, anti-inflammatory actions occur with some other lipoxygenase-derived eicosanoids. Lipoxins (LXA4 and LXB4) stop PMN infiltration and reduce expression of inflammatory cytokines by microglia cells [66]. LXA4 directly interacts, for example, with human FPRL1 (ALX/FPR2) and cysLT1 receptors, as well as affecting receptors for LTB1, cytokine/chemokines (e.g., TNF) and growth factors (*i.e.*, VEGF) in human leukocytes, vascular cells and mucosal epithelial cells [66]. All of these contribute to regulate the resolution of inflammation [66]. In parallel with other eicosanoids, oxidation of the 15-hydroxyl group essentially inactivates lipoxin’s ability to stop PMN transmigration or to reduce cell adhesion. Additionally, *n*-3 EFA-derived bioactive mediators, resolvins and protectins, act in a programmed resolution of inflammatory conditions. 

Some cytochrome P-450 (CYP) enzymes can form active eicosanoids. The CYP epoxygenase enzymes make epoxyeicosatrienoic acids (EETs) that have anti-hypertensive actions and are anti-inflammatory, whereas CYP hydroxylase enzymes generate HETEs, such as 20-HETE, that have cardiovascular and proinflammatory activities [67]. Epoxide-induced relaxation of vascular smooth muscle is counteracted by epoxide hydrolase action. Much effort is now directed to identifying epoxide receptors and developing drugs that inhibit the hydrolase and strengthen the beneficial epoxide-induced relaxation that diminishes hypertension [68]. 

Different groups in the biomedical community focus attention on different healthful and harmful effects of EFA-based tissue actions. The examples noted above illustrate the wide range of *n*-3 and *n*-6 mediators that contribute to varying proportions of the overall health risk and are measured in very different ways with different metrics. The overall beneficial and harmful consequences of the diverse EFA-based actions on physical and mental health are too complex to estimate any single outcome. In the end, an unequivocal consequence of eating *n*-3 and *n*-6 nutrients in our daily foods may be objectively reflected in firm endpoints like deaths or the actual annual healthcare claim costs [13]. 

## 4. Preventing the Consequences of EFA Imbalances

### 4.1. Omega 3 Fatty Acids in Cardiovascular Health

The signs and symptoms that determine the presence of a health disorder give a focal point to mobilize resources for remedial action. However, too narrow a focus on the signs may divert attention from early preventable causes of the signs [13]. Without adequate prevention, cardiovascular disease (CVD) now causes one in three (approximately 800,000) deaths reported each year in the United States, and approximately half of the U. S. adult population has one or more of the preventable risk factors for CVD [69]. The USA healthcare costs from CVD alone are estimated at $444 billion [70], and CVD prevalence and costs are projected to increase substantially [70,71]. Creating a sense of improved health by treating only signs and symptoms without removing the nutrient conditions that cause them (and continues to impair health and cause further financial losses) might be regarded by some people as inefficient and unethical. New more effective prevention strategies are needed to limit the growing burden of CVD [70]. 

The introduction to this review included CVD with a very wide range of health disorders implicated in relative deficits of *n*-3 HUFA and excessive actions of *n*-6 mediators. Recent changes in food production and marketing have created an environment that has a greater supply of *n*-6 nutrients than in the past [72]. The consequence of eating imbalanced proportions of *n*-3 and *n*-6 EFA is to develop imbalanced proportions of HUFA-based bioactive mediators of human physiology [26]. A simple primary prevention program [13,73] could inform people of their personal blood HUFA proportions [13,25] and of Omega 3–6 Balance Scores of foods [40,41]. The information could stimulate voluntary food choices that decrease the proportion of *n*-6 in tissue HUFA and decrease the financial losses from chronic actions of *n*-6 mediators [13,73]. 

### 4.2. Focusing on Mediators in Preventable Disorders

**Figure 3:**
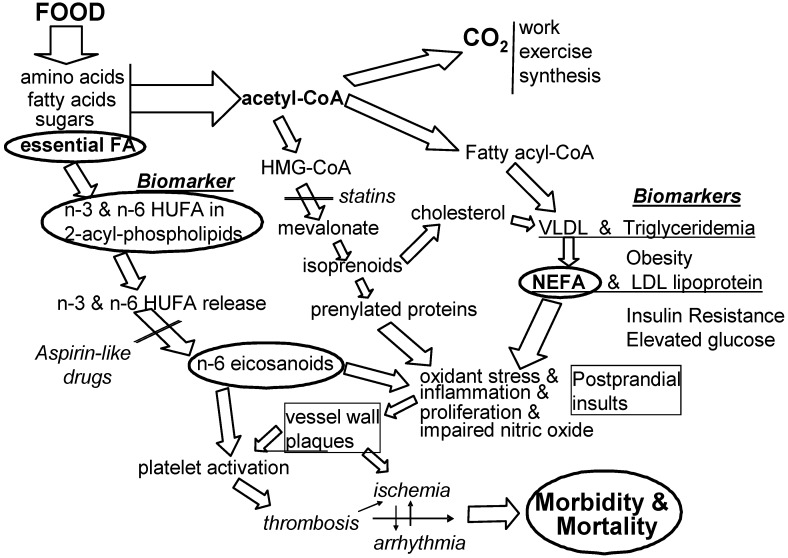
Associated predictive risk factors and causal mediating risk factors. Among the many conditions associated with developing cardiovascular morbidity and mortality, one is imbalanced intakes of *n*-3 and *n*-6 EFA (shown at the left) and another is too many calories per meal (noted at the center). Large clinical trials have been designed to lower some calorie-related biomarkers associated with health risk (noted at the right).

There is wide agreement that all risk factors can predict (to differing degrees) the likelihood of an impending healthcare problem. There is also wide agreement that not all predictive risk factors are causal factors. Figure 3 shows the context of molecular events that link voluntary food choices to preventable signs and symptoms of CVD. The EFA-based causal events on the left side of the figure illustrate how omega-3 fatty acids aid cardiovascular health as described in detail in earlier parts of this review. Intervention will likely be more effective when it prevents causal rather than non-causal conditions.

For six decades, the biomedical community has pursued a concern that cardiovascular disease (CVD) is caused in part by eating too much energy-dense food [6]. We know that when intake of food energy is much faster than can be “burned” in the next few hours, the liver converts the excess into fatty acids and cholesterol and secretes them into the blood in the form of very low density lipoproteins (VLDL). Elevated plasma triglycerides (triglyceridemia) have long been viewed as a strong predictive risk factor for CVD morbidity [74]. The triglycerides in circulating VLDL are hydrolyzed to non-esterified fatty acids (NEFA) which exert irritating, inflammatory effects on mammalian cells and induce insulin resistance [75] (especially when tissues have more *n*-6 HUFA than *n*-3 HUFA). As NEFA is released from circulating VLDL triglycerides, the resulting low density lipoproteins (LDL) appear in plasma. Although both NEFA and LDL are produced together, most attention has been on the cholesterol in the associated circulating LDL [6]. The role of NEFA remains neglected in most discussions and hypotheses of mediators of CVD, although inflammatory events are increasingly acknowledged as mediators [76,77]. Perhaps a clearer focus on how the proportions of *n*-6 HUFA in tissue HUFA mediate inflammatory and thrombotic events will lead to more controlled studies of the diet-based mediators noted in Figure 3.

Results from a 25-year follow-up of one of the early epidemiological studies on CVD suggest that the food energy imbalances which elevate blood triglycerides and cholesterol may become fatal only to the degree that the tissue environment contains more *n*-6 HUFA than *n*-3 HUFA [6,78]. For example, a large study in Japan (where the food environment has maintained more equal proportions of *n*-3 and *n*-6 HUFA) showed blood cholesterol was not associated with mortality for these people [79]. It is possible that the NEFA that must be released whenever LDL-cholesterol is formed is promoting vascular inflammation which is amplified in tissues that have high proportions of *n*-6 in HUFA [6]. This view reconciles the observed predictive association of circulating LDL with the occurrence of CVD for populations with high proportions of *n*-6 HUFA without requiring that LDL is a mediator. 

Recent evidence for a role of dietary linoleic acid (18:2*n*-6) in causing CVD risk [80] creates a paradox with the long-standing belief that dietary 18:2*n*-6 gives cardiovascular benefits by lowering circulating levels of LDL cholesterol. Some groups have urged the public to eat at least 5%–10% of energy as 18:2*n*-6 to lower blood cholesterol levels and hopefully reduce risk of CVD [81,82]. Nevertheless, the evidence cited in this review suggests that the overall consequence of decreasing intakes of *n*-6 nutrients and increasing intakes of *n*-3 nutrients may give beneficial reductions in CVD and overall healthcare costs. Increased inflammatory action occurs when *n*-6 eicosanoids act on EP1, FP and TP receptors and amplify the signaling mediated by the prenylated protein, Rho [56]. When statin drugs lower the availability of activating isoprenoids and when tissue HUFA have higher proportions of competing *n*-3 HUFA (see Figure 3), the pro-inflammatory action of Rho is decreased.

Some efforts to lower the total daily calorie intake (and lower body mass index or obesity) may not lower the transient postprandial rise in NEFA that comes from eating a large meal. As a result, repeated transient postprandial vascular insults may be amplified into chronic inflammatory harm by the mediating actions of prenylated proteins and *n*-6 eicosanoids. Also, some efforts to lower blood glucose that has been elevated by NEFA-induced insulin resistance may not lower the associated *n*-6 eicosanoid-enhanced chronic inflammatory events that lead to CVD morbidity and mortality (see Figure 3). The greater action of *n*-6 LTB4 than *n*-3 LTB5 with the BLT-1 receptor [64] is an important aspect of how food choices can influence inflammation and insulin resistance and elevate blood glucose. Efforts to decrease associated risk factors that are not causal risk factors may be less effective than decreasing causal risk factors when trying to lower CVD. Eating more omega-3 EFA and less omega-6 EFA to alter the inner environment of cells and tissues [13,73] seems a likely useful preventive nutrition action for lowering CVD incidence and prevalence.

## 5. Conclusions

Considerable evidence shows that dietary EFA form hormone-like mediators that act on receptors and affect the physiology of many cells and tissues. Evidence also indicates that excessive *n*-6 signaling actions may cause many chronic physical and mental health disorders. Effective preventive nutrition interventions may help people choose foods to develop a balance of *n*-3 and *n*-6 HUFA in their tissues in a way that may lower the severity of the disorders and lower their personal risk for healthcare expenses.
